# Surgical repair of esophageal atresia: do trans-anastomotic feeding tubes have an impact on the outcome?

**DOI:** 10.3389/fped.2025.1685089

**Published:** 2025-11-13

**Authors:** Mohamed Yousef Batikhe, Mostafa Redwan, Ahmed Gafar, Mohamed Ramadan

**Affiliations:** Faculty of Medicine, Sohag University, Sohag, Egypt

**Keywords:** esophageal atresia/tracheoesophageal fistula, EA/TEF, trans anastomotic feeding tubes, anastomotic leak, anastomotic stricture

## Abstract

**Introduction:**

Transanastomotic feeding tubes (TATs) are placed to allow early feeding following esophageal atresia/tracheoesophageal fistula (EA/TEF) repair. However, recently these tubes were linked to increased rates of postoperative complications. The aim of this study is to report our single center experience with and without the use of TATs in patients with EA/TEF.

**Patients and methods:**

The data of 152 patients operated for EA and distal TEF in our hospital from January 2014 to September 2024 were retrospectively reviewed. Patients were divided into two groups; those who did not have TATs (NOTAT group) and those who have their TATs left in-place until deliberately removed according to postoperative care protocol (TAT group). Both groups were compared regarding baseline characteristics, operative findings, postoperative care and postoperative complications.

**Results:**

The TAT group included 130 patients while the NOTAT group included 22 patients. There were no statistically significant differences between the two groups in terms of birth weight, gestational age, the rate of prematurity or the associated anomalies. The time of commencing enteral feeding was significantly earlier in the TAT group (median POD #3 vs. POD# 13 in the NOTAT group) and the duration of TPN was significantly shorter (mean of 6.6 vs. 16.2 days in the NOTAT group). There were no statistically significant differences between the two groups regarding the rate of postoperative anastomotic leaks (23.0% in the TAT group vs. 18.1% in the NOTAT group; *p* = 0.700) or strictures.

**Conclusion:**

Trans anastomotic feeding tubes (TATs) offer benefits in terms of early enteral feeding, and shorter duration of parenteral nutrition for patients with EA/TEF undergoing surgical repair. At the same time, they don't seem to add a risk of anastomotic leaks or strictures. Large prospective randomized studies are required to further evaluate any possible relationships between TATs and the postoperative complications following repair of EA/TEF.

## Introduction

Esophageal atresia (EA) is one of the most challenging surgical conditions in neonates. Although the postoperative mortality has decreased over the past 30 years, the postoperative complications are still significant (1). Recently, there has been an ongoing debate regarding the potential benefits vs. the risks associated with the use of transanastomotic tubes (TATs) in the surgical repair of EA/TEF. Proponents argue that TATs facilitate early postoperative enteral feeding, reduce the need for prolonged parenteral nutrition, and help maintain anastomotic patency. However, several studies have suggested that TATs may contribute to an increased incidence of anastomosis-related complications, particularly anastomotic leak and anastomotic stricture (2, 3).

The purpose of this study was to determine whether the presence of TATs influences postoperative outcomes after surgical repair of EA/TEF.

## Patients and methods

We conducted a retrospective single-center review of all consecutive infants with esophageal atresia (EA) treated between January 2014 and September 2024. Only infants with EA and a distal tracheoesophageal fistula (TEF) (Gross type C) who underwent primary esophageal anastomosis during the initial surgery were included. Infants who were identified as having a long-gap EA requiring staged repairs were excluded from the study. Patients who died within 48 h post-operatively from unrelated causes and those who were lost to follow-up or those with incomplete medical records were also excluded from the study.

### Data collection and analysis

After obtaining approval from the institutional review board (IRB), data examined included preoperative patients' characteristics, namely sex, birth weight, gestational age, prematurity, and the presence of associated anomalies. Data related to the operation and postoperative care included the age at surgical repair, the technique of esophageal anastomosis, anastomosis under tension, the use of trans-anastomotic feeding tubes (TATs), the time of commencing enteral feeding (tube/oral), and the duration of parenteral nutrition. Early postoperative data included the occurrence of anastomotic leaks, sepsis, pneumonia, the total length of hospital stay, and all-cause in-hospital mortality. Data on follow up after discharge from the hospital included the occurrence of anastomotic strictures and gastroesophageal reflux (GER).

The primary outcome parameters were anastomotic leaks and anastomotic strictures. Secondary outcomes included the time to start enteral feeding and the duration of TPN. Anastomotic leaks were suspected by the passage of large amounts of saliva through the chest tubes and confirmed on contrast esophagogram. Anastomotic strictures were suspected when patients developed dysphagia or regurgitation after starting oral feeding and confirmed by a contrast esophagogram.

Patients were divided into two groups based on the presence or absence of TAT in the postoperative period. Those with TAT were termed “TAT group” and those with no TAT were termed “NOTAT group”.

Statistical analysis was conducted by a statistician using the chi-squared test, Fisher's exact test, Student's *t*-test, and two-proportion z-test as applicable. A *p*-value of less than 0.05 was considered statistically significant.

### Surgical technique and postoperative care protocol

In our center, the surgical repair of EA with distal TEF is done through a right posterolateral thoracotomy using either an extra pleural or intrapleural approach. The tracheoesophageal fistula is divided and closed using 2–3 interrupted sutures followed by a single layer end-to-end esophageal anastomosis over a 6 French nasogastric tube using full-thickness interrupted 5/0 Vicryl® sutures. A chest tube is routinely placed. The decision to insert or not to insert a TAT was left to the operating surgeon.

Enteral feeding is often commenced on the second or third postoperative day via the TAT. If TAT was not inserted, the patient is kept on total parenteral nutrition (TPN) for 7–10 days, and then evaluated for the presence of any anastomotic leaks initially through observing the output from the chest tube and then by performing a contrast esophagogram before starting oral feeding.

## Results

A total of 186 patients with esophageal atresia and distal tracheoesophageal fistula were treated during the study period. Of these, 152 patients fulfilled the inclusion criteria and were included in the study (Table 1).

**Table 1:** Summary of the results.

Parameter	All (*n* = 152)	TAT (*n* = 130)	NOTAT (*n* = 22)	*p* value
Baseline characteristics				
Males; *n* (%)	95/152 (62.8%)	86/130 (66.1%)	9/22 (40.9%)	0.003
BW (gm); mean ± SD (range)	3,250 ± 230 (1,900–3,450)	3,350 ± 250 (2,000–3,450)	3,100 ± 220 (1,900–3,200)	0.061
GA (wk); mean ± SD (range)	39.4 ± 2.1 (31–42)	38.8 ± 1.9 (31–40)	39.2 ± 2.3 (33–42)	0.447
Preterm infants; *n* (%)	35/152 (23.0%)	30/130 (23.0%)	5/22 (22.7%)	0.971
Associated anomalies; *n* (%)	45/152 (29.6%)	39/130 (30.0%)	6/22 (27.2%)	0.421
Operation and postoperative care				
Age at repair (d); median (range)	4 (1–9)	5 (1–9)	4 (1–7)	0.294
Anastomosis under tension; *n* (%)	27/152 (17.7%)	24/130 (18.4%)	3/22 (13.6%)	0.520
Commencing enteral feeds (POD#); median (range)	5 (2–18)	3 (2–18)	13 (10–17)	0.001
TPN (d); mean ± SD (range)	10.8 ± 4.6 (4–28)	6.6 ± 2.3 (4–18)	16.2 ± 3.3 (11–28)	0.0003
Early postoperative outcomes (in-hospital)				
Hospital stays (d); median (range)	18 (4–32)	15 (4–24)	18 (8–32)	0.147
Anastomotic leak; *n* (%)	34/152 (22.3%)	30/130 (23.1%)	4/22 (18.1%)	0.700
Reoperation; *n* (%)	10/152 (6.5%)	9/130 (6.9%)	1/22 (4.5%)	0.722
Sepsis; *n* (%)	33/152 (21.7%)	29/130 (22.3%)	4/22 (18.1%)	0.700
Pneumonia; *n* (%)	60/152 (39.4%)	49/130 (37.6%)	11/22 (50%)	0.300
In-hospital all-cause mortality; *n* (%)	42/152 (27.6%)	36/130 (27.6%)	6/22 (27.2%)	0.935
Late postoperative outcomes (post-discharge)				
Follow up period (m); mean ± SD (range)	22 ± 2.6 (6–50)	25 ± 2.3 (6–38)	19 ± 3.1 (11–50)	0.070
Anastomotic stricture; *n* (%)	39/110 (35.4%)	34/94 (36.1%)	5/16 (31.2%)	0.610
GER; *n* (%)	49/110 (44.5%)	42/94 (44.6%)	7/16 (43.7%)	0.940

n, number; %, percentage; GA, gestational age; BW, birth weight; TPN, total parenteral nutrition; wk, weeks; gm, grams; d, days; m, months; SD, standard deviation; POD#, postoperative day number; GER, gastroesophageal reflux.

Among the 152 patients, 22 patients (14.5%) had no TAT (NOTAT group). The remaining 130 patients (85.5%) had their TAT in place until deliberately removed according to the postoperative care protocol (TAT group).

### Baseline characteristics

A higher percentage of males was observed in the study population (95/152, 62.8%), with a statistically significant difference between the TAT and NOTAT groups (66.1% vs. 40.9% respectively (*p* = 0.003).

The mean birth weight for all patients in the study was 3,250 ± 230 g (1,900–3,450 g). The birth weight was higher in the TAT than the NOTAT patients (3,350 ± 250 vs. 3,100 ± 220 g respectively). However, the difference was not statistically significant (*p* = 0.061).

The mean gestational age was 39.4 ± 2.1 weeks (31–42 weeks). Thirty-five infants (23.0%) were preterm (born at <37 weeks). The mean gestational age was 38.8 ± 1.9 (31–40) in the TAT group and 39.2 ± 2.3 (33–42) in the NOTAT group; a difference that was not statistically significant (*p* = 0.447). Both groups had nearly similar proportions of preterm infants (23.0%, 22.7% in the TAT and NOTAT groups respectively).

### Associated anomalies

Forty-five infants (29.6%) had associated anomalies; 13 (8.5%) had cardiac anomalies, 9 (5.9%) had anorectal anomalies, 8 (5.2%) had renal anomalies, 6 (3.9%) had chromosomal anomalies (trisomy 21), 6 (3.9%) had limb abnormalities, and 3 (1.9%) had duodenal anomalies. The rate of associated anomalies was similar in both groups (30.0% and 27.2% for TAT and NOTAT groups respectively, *p* = 0.421).

### Operation and postoperative care

The surgical technique was constant throughout the study period with no major modifications. The median age at the time of repair for the whole study population was 4 days (1–9), and it was not significantly different between the two groups (TAT = 5 days, and NOTAT = 4 days, *p* = 0.294).

Anastomosis under tension was reported in 17.7% of all patients in the study (27/152). There was no significant difference in the rate of anastomosis under tension between the 2 groups as it was 18.4% (24/130) in the TAT group, and 13.6% (3/22) in the NOTAT group (*p* = 0.520).

Enteral feeds commenced at a median of 5 days postoperatively (2–18) for the whole study population. Enteral feeds commenced earlier in infants of the TAT group (median POD# 3) compared to the NOTAT group (median POD# 13), a difference that was highly significant (*p* = 0.001). The duration of total parenteral nutrition (TPN) was also significantly (*p* = 0.0003) shorter in the TAT group (mean of 6.6 ± 2.3 days) compared to the NOTAT group (mean of 16.2 ± 3.3 days).

### Early postoperative outcomes (in-hospital)

The overall median hospital stay was 18 days (4–32). For the TAT group it was 15 days (4–24), and for the NOTAT group was 18 days (8–32), a difference that was statistically non-significant (*p* = 0.147).

Anastomotic leaks occurred in 22.3% (34 out of 152) and reoperation was required in 6.5% (10 out of 152) of all patients. The anastomotic leak rate was higher in the TAT group (23.0%; 30 out of 130) than in the NOTAT group (18.1%; 4 out of 22). However, this difference was not statistically significant (*p* = 0.700). Similarly, the rate of reoperation was higher in the TAT (6.9%; 9 out of 130) than in the NOTAT (4.5%; 1 out 22) groups but the difference was not statistically significant (*p* = 0.722).

Variables examined by univariate analysis for an association with anastomotic leak (Table 2) included prematurity, associated anomalies, anastomosis under tension, TAT use, reoperation and birth weight. None of these variables was significantly associated with the development of anastomotic leak including TAT use with an odd ration (OR) of 1.35 (95% CI 0.36–4.96, *p* = 0.705). In multivariate analysis, after adjusting for these factors, still no significant association was found between the use of TAT and anastomotic leak (OR of 1.75; 95% CI 0.16–5.37, *p* = 0.621).

**Table 2:** Variables examined by univariate analysis for an association with anastomotic leak.

Variable	Anastomotic leak rate	Odds ratio (95% CI)	*p* value
Preterm infants	25.7%	1.27 (0.58–3.80)	0.541
Associated anomalies	15.6%	0.61 (0.26–2.41)	0.252
Anastomosis under tension	22.2%	1.38 (0.52–3.61)	0.522
TAT	23.1%	1.35 (0.36–4.96)	0.705
Reoperation	20.0%	1.20 (0.24–5.83)	0.824
Birth weight (gm); mean ± SD	3,219 ± 240	-	0.481

The overall sepsis rate was 21.7% in the study population (33 out of 152). The sepsis rate in the TAT patients was higher than in the NOTAT patients (22.3%; 29 out of 130 vs. 18.1%; 4 out of 22). Moreover, pneumonia was reported in 39.4% (60 out of 152) overall, with a lower rate of 37.6% (49 out of 130) sepsis in the TAT patients than in the NOTAT patients (50%; 11 out of 22). However, neither of these differences in the sepsis rate nor the pneumonia rate was statistically significant (*p* = 0.700 and 0.300 respectively).

The overall in-hospital all-cause mortality rate was 27.6% (42 out of 152) leaving 110 patients (72.4%) for intermediate- and long-term follow up. In-hospital deaths occurred in 27.6% of patients (36 out of 130) in the TAT group and 27.2% of patients (6 out of 22) in the NOTAT group; a nearly equal rate in both groups with no statistically significant difference (*p* = 0.935).

### Late postoperative outcomes

One hundred and ten patients were discharged alive from the hospital and were followed up for a mean period of 22 ± 2.6 months (range 6–50). Among them, 94 (85.4%) belonged to the TAT group and were followed-up for a mean period of 25 ± 2.3 months (range 6–38) and 16 (14.5%) belonged to the NOTAT group and were followed up for a mean period of 19 ± 3.1 months (range 11–50).

Out of these 110 patients, 39 (35.4%) developed symptomatic anastomotic strictures. The stricture rate was higher in the TAT group (36.1%; 34 out of 94) than in the NOTAT group (31.2%; 5 out of 16), although this difference was not statistically significant (*p* = 0.610). Meanwhile, gastroesophageal reflux (GER) was diagnosed in 49 out of these 110 patients (44.5%). Again, the difference in GER rates between the TAT and NOTAT groups was not statistically significant (44.6%; 42 out of 94 vs. 43.7%; 7 out of 16 respectively; *p* = 0.940).

Variables examined by univariate analysis for an association with anastomotic stricture (Table 3) included prematurity, associated anomalies, anastomosis under tension, TAT use, reoperation and birth weight. None of these variables was significantly associated with the development of anastomotic stricture including TAT use with an odd ration (OR) of 1.72 (95% CI 1.35–5.74, *p* = 0.610]. In multivariate analysis, after adjusting for these variables, still no significant association was found between the use of TAT and the development of anastomotic stricture (OR of 1.55; 95% CI 0.26–4.36, *p* = 0.711).

**Table 3:** Variables examined by univariate analysis for an association with anastomotic stricture^a^

Variable	Anastomotic stricture rate	Odds ratio (95% CI)	*p* value
Preterm infants	28.6%	1.35 (0.63–2.90)	0.432
Associated anomalies	24.4%	0.72 (0.33–3.59)	0.421
Anastomosis under tension	29.6%	1.52 (0.61–3.82)	0.373
TAT	36.1%	1.72 (1.35–5.74)	0.610
Reoperation	20.0%	1.18 (0.25–5.46)	0.824
Birth weight (gm); mean ± SD	3,241 ± 260	-	0.251

a Results from 110 survivors after excluding 42 in-hospital mortality patients.

## Discussion

Esophageal atresia (EA)/tracheoesophageal fistula (TEF) is a rare malformation with a prevalence of 1 per 3,500 births (4). Mortality rates after surgical repair of these anomalies ranged from as low as 7.2% in high-income countries to as high as 85.7% in low-income countries (5). Survival rates up to 95% after surgical repair have also been reported in specialized centers (6). The mortality rate in our cohort is relatively high (27.6%). This may reflect differences in case mix, access to neonatal intensive care, and prevalence of associated anomalies. Nonetheless, the mortality rates in both groups (TAT and NOTAT) are nearly equal.

In recent years, however, the emphasis in treating infants with esophageal atresia has shifted from solely ensuring survival to improving other key outcomes, reducing morbidities, minimizing the burden of care and optimizing resource utilization (7).

Transanastomotic tubes (TATs) are often placed to allow early feeding in patients following EA/TEF repair. In the absence of a gastrostomy or other routes for enteral feeding, parenteral nutrition is almost obligatory for the critical first week after repair of esophageal atresia. The concept of trans anastomotic feeding is elimination of this waiting period while avoiding the complications of central venous catheters and parenteral nutrition (8–11). The results of our study support this concept as the commencement of enteral feeding was significantly earlier in the TAT group and the duration of TPN was significantly shorter. Moreover, there was a trend toward shorter hospital stay in the TAT group, though this did not reach statistical significance. These findings emphasize the pragmatic benefits of TATs in resource-limited settings, where prolonged parenteral nutrition contributes substantially to cost, infection risk, and length of stay.

Theoretically TATs have the potential benefit of reducing anastomotic complications such as anastomotic leaks and stricture by stenting the anastomosis. However, concern persists regarding the possibility that a TAT may mechanically impair anastomotic healing, increase local ischemia, or serve as a nidus for inflammation and fibrosis, predisposing to anastomotic complications namely leakage and stricture formation (7).

The effect of transanastomotic tubes on the healing of esophageal anastomosis was evaluated in two interesting animal studies, although with contrasting results. Carachi et al. found no significant adverse effect of tube placement on the anastomotic lumen in an animal model (12). Conversely, Yurtcu et al. observed reduced bursting pressure and lumen diameter in rabbits with a retained nasogastric tube (13).

Clinical studies have also yielded contrasting conclusions. Our results did not demonstrate any statistically significant association between TAT use and either anastomotic leak or stricture formation. Similar findings were reported by other single-institution reviews. Alabbad et al. (11) and Narayanan et al. (14) both concluded that TATs do not independently increase the risk of anastomotic complications. In a recent study by Morsi and Misra, there were no anastomotic leaks and only 20% stricture rates in spite of using transanastomotic tubes in all patients (15).

By contrast, two large multicenter studies have suggested that TAT use might be an independent predictor of anastomotic stricture. Lal et al. (2) and LaRusso et al. (3) both found a significant association between TAT use and subsequent stricture formation, even after adjusting for confounders such as gestational age, birth weight, anastomotic tension, and leak. A meta-analysis by Wang et al. (16) further supported this observation, reporting a pooled odds ratio indicating higher stricture risk in patients with TATs, though no difference in leak rates. Another large single-institution review by Fusco et al. reported higher anastomotic stricture rates with the use of TAT, however there was no increase in the leak rate (17).

The discrepancy between those studies and the present one, probably represents differences in patient populations in different institutions, postoperative management protocols, and tube care. Specifically, in our center the surgical technique ensures placement of the tube so that the side openings are away from the anastomotic line to avoid friction, the dwell time is kept at minimum (immediate tube removal after adequate enteral feeding has been achieved), and the feeding protocol that strictly minimizes traction or pressure on the tube and subsequently on the anastomotic suture line.

## Limitations

There are several important limitations to this study that need to be mentioned. First, this is a retrospective single-center study, and therefore it carries an inherent risk of selection bias and reduced ability to determine causality. In addition, some important details were poorly documented in the patients’ records including the criteria for deciding whether or not to insert a TAT, the timing of extubation, the duration of paralysis, and preoperative prophylaxis and management of GERD. Second, the sizes of the two groups were not equal, with significantly fewer patients in the NOTAT group. This reflects real clinical practice in our unit over the study period; however it limited proper statistical analysis. Although multivariate regression analysis is ideal to adjust for potential confounders and identify independent predictors of anastomotic complications, the relatively small size of the NOTAT group makes multivariate analysis underpowered. Third, the postoperative feeding practices were not the same between groups, since early enteral feeding was intrinsic to TAT use, a confounding influence that cannot be fully controlled in this design. Finally, the relatively high in-hospital mortality rate (27%) may have affected the long-term outcomes, as some of those patients who did not survive would have developed complications if they survived.

## Conclusions

While the results of our study suggest that transanastomotic tubes allow earlier enteral feeding and reduced TPN duration and at the same time they don't increase the stricture and leak rate, other larger multicenter retrospective studies suggested that they increase the risk of anastomotic strictures. An ongoing multi-center randomized clinical trial (NCT03730454) is investigating whether the use of a transanastomotic tube during the repair of esophageal atresia affects the occurrence of symptomatic anastomotic strictures requiring dilation within 12 months (18). The study has finished recruiting and its results are awaited. The results of this trial should shed light on this controversy and aid the decision of whether or not to insert a transanastomotic tube at the time of EA/TEF repair.

## Data Availability

The original contributions presented in the study are included in the article/Supplementary Material, further inquiries can be directed to the corresponding author.
